# Identification of WDR74 and TNFRSF12A as biomarkers for early osteoarthritis using machine learning and immunohistochemistry

**DOI:** 10.3389/fimmu.2025.1517646

**Published:** 2025-01-28

**Authors:** Yiwei Chen, Jiali Lin, Detong Shi, Yu Miao, Feng Xue, Kexin Liu, Xiaotao Wang, Changqing Zhang

**Affiliations:** ^1^ Department of Orthopedics, Shanghai Sixth People’s Hospital Affiliated to Shanghai Jiao Tong University School of Medicine, Shanghai, China; ^2^ Institute of Microsurgery on Extremities, Shanghai Sixth People’s Hospital Affiliated to Shanghai Jiao Tong University School of Medicine, Shanghai, China; ^3^ Obstetrics and Gynecology Hospital, Institute of Reproduction and Development, Fudan University, Shanghai, China

**Keywords:** osteoarthritis, ubiquitination, machine learning, single-cell RNA sequencing, diagnosis

## Abstract

**Background:**

Osteoarthritis (OA) is a chronic joint condition that causes pain, limited mobility, and reduced quality of life, posing a threat to healthy aging. Early detection is crucial for improving prognosis. Recent research has focused on the role of ubiquitination-related genes (URGs) in early OA prediction. This study aims to integrate URG expression data with machine learning (ML) to identify biomarkers that improve diagnosis and prognosis in the early stages of OA.

**Methods:**

OA single-cell RNA sequencing datasets were collected from the GEO database. Single-cell analysis was performed to investigate the composition and relationships of chondrocytes in OA. The potential intercellular communication mechanisms were explored using the CellChat R package. URGs were retrieved from GeneCards, and ubiquitination scores were calculated using the AUCell package. Gene module analysis based on co-expression network analysis was conducted to identify core genes. Additionally, ML analysis was performed to identify core URGs and construct a diagnostic model. We employed XGBoost, a gradient-boosting ML algorithm, to identify core URGs and construct a diagnostic model. The model’s performance was evaluated using the area under the curve (AUC) of the receiver operating characteristic (ROC) curve. In addition, we explored the relationship between core URGs and immune processes. The ChEA3 database was utilized to predict the transcription factors regulated by core ubiquitination-related genes. The expression of select URGs was validated using qRT-PCR and immunohistochemistry (IHC).

**Results:**

We identified WDR74 and TNFRSF12A as pivotal ubiquitination-related genes associated with OA, exhibiting considerable differential expression. The diagnostic model constructed with URGs exhibited remarkable accuracy, with area under the curve (AUC) values consistently exceeding 0.9. The expression levels of WDR74 and TNFRSF12A were significantly higher in the IL-1β-induced group in an *in vitro* qPCR experiment. The IHC validation on human knee joint specimens confirmed the upregulation of WDR74 and TNFRSF12A in OA tissues, corroborating their potential as diagnostic biomarkers.

**Conclusions:**

WDR74 and TNFRSF12A as principal biomarkers highlighted their attractiveness as therapeutic targets. The identification of core biomarkers might facilitate early intervention options, potentially modifying the illness trajectory and enhancing patient outcomes.

## Introduction

Osteoarthritis (OA) is a degenerative joint disease affecting over 250 million people worldwide, leading to significant physical disability and substantial societal burden (1–5). The onset and progression of OA are complex, posing challenges for clinicians and researchers (3, 6, 7). OA development is linked to inflammatory, mechanical, and metabolic processes, leading to the deterioration of structures such as articular cartilage, subchondral bone, synovium, joint capsule, ligaments, and peri-articular muscles (8, 9). However, medical professionals often face challenges in proactive monitoring and managing OA in its early stages (10–12). Consequently, patients typically present with symptoms during the intermediate and advanced stages, limiting effective disease management (13, 14). Therefore, it is crucial to identify and validate prospective biomarkers for early OA detection. This could enable more proactive management and improve patient outcomes (15).

Recent genomic breakthroughs have shed light on the complex molecular landscape of OA, highlighting the ubiquitin-proteasome system (UPS) as a crucial regulatory mechanism in the disease’s pathogenesis (16, 17). A growing contingent of researchers is concentrating on the influence of the ubiquitination process in OA, rendering it a prominent area of investigation. Ubiquitination is a crucial post-translational modification that plays a role in protein breakdown and cellular communication, and it is impaired in several disorders, including OA (16, 18). This process entails the conjugation of ubiquitin molecules to target proteins, which is essential for controlling protein stability, function, and intracellular localization (17). Ubiquitination influences OA by contributing to the deterioration of articular cartilage, involvement in immune system inflammatory responses, and modulation of apoptosis. Furthermore, ubiquitination affects the function and destiny of chondrocytes by engaging with other cellular signaling networks, including the Wnt/β-catenin pathway (19). Additionally, ubiquitination can influence the activity of transcription factors, thereby modulating the expression of genes related to OA (20). Consequently, therapies aimed at the ubiquitination process may yield novel techniques for OA treatment, and the identification of differentially expressed ubiquitination-related genes (URGs) in OA could present new biomarkers for diagnosis and therapy.

Advances in genomic technology, particularly single-cell RNA sequencing (scRNA-seq), have significantly enhanced our understanding of cellular diversity in complex tissues like cartilage (21, 22). ScRNA-seq enables the precise identification of various cell types and their unique molecular characteristics within the joint, offering detailed, high-resolution gene expression profiles (23). However, the extensive and complex data generated by these technologies require advanced analytical methods to fully understand their biological significance. The advent of machine learning (ML) has revolutionized bioinformatics, enabling the precise and effective analysis of complex biological datasets (24, 25). ML algorithms can detect patterns and connections in large gene expression datasets, uncovering diagnostic indicators that might not be identifiable with conventional statistical methods (26–28).

In this study, we present a novel diagnostic approach that integrates bioinformatics and advanced ML algorithms. Our method leverages the high resolution of scRNA-seq to identify the diverse cell types in cartilage tissue. Meanwhile, we used robust bioinformatics techniques, including quality control, dimensionality reduction, and cell type annotation, to ensure data accuracy and clarity. By applying ML, we aimed to identify patterns and connections in gene expression data that conventional statistical methods might miss. Finally, we identified key cell types and hub genes and validated these key cell types and hub genes through ML analysis and clinical samples. Additionally, we developed a diagnostic model to enhance the application of hub genes for OA diagnosis. Furthermore, we investigated the relationship between hub genes and immune cells, immunological processes, and immune checkpoint molecules. We also illustrated the association between hub gene expression with the TNF family and the chemokine family. In the research, we stressed that the occurrence and progression of OA are significantly influenced by genes associated with ubiquitination. This study identifies diagnostic indicators connected with ubiquitination-related genes for patients, improving the capacity for early-stage OA diagnosis. It provides novel insights for individualized medical diagnosis and therapeutic approaches.

## Methods

### Patients and specimens

The clinical sample data source utilized in this investigation was received from Shanghai Sixth People’s Hospital. Articular specimens were procured from 55 patients who were diagnosed with knee OA and underwent knee arthroplasty surgery. Specimens from all layers of cartilage and subchondral bone were separately obtained and separated into two groups: the damaged area (designated as OA) and the corresponding undamaged area (designated as undamaged). All human studies were approved by the ethics committee of Shanghai Sixth People’s Hospital [Approval Number: 2019-KY-007(K)], and full written consents were obtained before the operative procedure. We assessed the expression level of genes associated with core necroptosis in 55 OA tissues and 55 undamaged tissues. We performed all the experiments described in accordance with The Code of Ethics of the World Medical Association (Declaration of Helsinki).

### Data collection

The OA single-cell sequencing datasets were obtained from the GEO database (https://www.ncbi.nlm.nih.gov/geo/). Specifically, dataset GSE169454 was utilized, comprising three normal cartilage tissues and four OA cartilage tissues sequenced on the 10X sequencing platform. In addition to GSE169454, the analysis incorporated datasets GSE89406, GSE57218, and GSE117999. The GSE89406 dataset includes 28 normal samples and 22 OA samples, serving as the training set for the diagnostic model. Dataset GSE57218 consists of 7 normal samples and 33 OA samples, utilized as the initial validation set for the diagnosis model. Lastly, dataset GSE117999 comprises 10 normal samples and 10 OA samples, selected as the second validation set for the diagnostic model. These datasets were chosen to enhance the robustness and reliability of our analysis across different cohorts and conditions.

### Single-cell sequencing data processing

The single-cell data analysis was performed using Seurat version 4.2.2. Upon importing the datasets, quality control measures were applied to remove cells exhibiting mitochondrial or ribosomal proportions exceeding 10% or 40%, respectively, and cells with nFeature_RNA values below 500 or above 5000. Data normalization and scaling were achieved using the SCTransform function. Dimensionality reduction and clustering were conducted based on the first 15 principal components using the RunUMAP function to generate results. Cell annotation was carried out with SingleR in accordance with literature reports. Marker genes for each cell type were identified using the FindAllMarkers function. Doublet cells were identified and removed using the DoubletFinder function.

### Cell communication analysis

The “CellChat” R package (version 1.5.0) was used to reveal the fundamental processes of cell-to-cell communication in single-cell data. The createCellChat function was utilized to instantiate a CellChat object, while the aggregateNet function detailed the signals originating from each cell type. The netVisual_circle function was utilized to visually represent the extent and significance of cell-to-cell communication, whereas the netAnalysis_computeCentrality function deduced the input and output weights of certain signaling pathways.

### Ubiquitination scoring

A total of 1232 ubiquitination-related genes were gathered from the Genecard official website, specifically selecting genes with a relevance score greater than 5. These genes comprise a gene set that is associated with ubiquitination. The AUCell package was employed for ubiquitination scoring, with the AUCell_calcAUC function acting as the main scoring mechanism. The ggplot2 package’s violin plot was utilized to exhibit the scores.

### High-dimensional weighted gene co-expression network analysis

HdWGCNA extends the functionality of WGCNA specifically for analyzing single-cell data, employing a systems biology approach to identify correlations between genes from high-throughput gene expression data. The SetupForWGCNA function was used to instantiate a WGCNA object, facilitating the initial setup for analysis. Subsequently, the MetacellsByGroups function was employed to generate metacells information, providing insights into cellular groupings based on gene expression profiles. To identify gene modules, an analysis of the co-expression network was performed using a soft threshold. This process included computing module eigengenes to identify core genes within each module. The analysis was executed using the hdWGCNA program, which specializes in handling single-cell transcriptomic data.

### Differential expression and enrichment analysis

In our study, we conducted differential expression analysis using the Limma software package. Genes were selected for further analysis if they exhibited an absolute log fold change (logFC) of at least 1 and had an adjusted P-value below 0.05. We set the significance level (α) at 0.05, which corresponds to a 5% risk of a Type I error, or incorrectly rejecting the null hypothesis. The P-value represents the probability of obtaining a test statistic as extreme as, or more extreme than, the observed result under the assumption that the null hypothesis is true. A P-value of 0.05 or lower led us to conclude that the observed changes in gene expression were statistically significant, prompting us to reject the null hypothesis. For enrichment analysis, we used the ClusterProfiler package to identify biological terms that were overrepresented among the differentially expressed genes. This approach assessed whether the observed count of genes in specific pathways was significantly higher than what would be expected by random chance. Furthermore, to visualize the differential expression results, we generated volcano plots with ggplot2. Additionally, we used ggplot2 to create star plots for visualizing the outcomes of our enrichment analysis. These visualizations provide a clear and intuitive representation of our findings.

### XGBoost machine learning and diagnostic model construction

The ML investigation employed XGBoost, a highly efficient gradient-boosting implementation. The analysis utilized the XGboost package along with the DALEX and breakDown packages for result interpretation. Partial dependence plots were employed to assess the significance of genes, where higher values indicated greater importance. The rms package was utilized to optimize the selection of core genes for OA diagnosis and develop a diagnostic model. Visualization of the model was achieved using the replot program to create a nomogram. ROC curves for the diagnostic model were generated using the pROC and ROCR packages. Additionally, decision curve analysis (DCA) was conducted using the rmda package to assess the clinical utility of the diagnostic model. To optimize the performance of our XGBoost model, we conducted a systematic process of hyperparameter tuning. We employed a grid search method combined with cross-validation to identify the optimal set of hyperparameters. For model validation, we utilized a rigorous cross-validation technique. Specifically, we employed K-fold cross-validation with K set to 5. This method involves dividing the dataset into K subsets, training the model on K-1 of these subsets, and then validating it on the remaining subset. This process is repeated K times, with each subset serving as the validation set once. The performance metrics are averaged across all K folds to provide a comprehensive assessment of the model’s performance. Additionally, decision curve analysis (DCA) was conducted using the rmda package to assess the clinical utility of the diagnostic model.

### Immune-related analysis

The GSVA software and GSEABase package were used to compute relative enrichment scores for 29 immune cell types and immunological processes. Heat maps were generated to visualize the associations between core genes and immune cells, processes, as well as checkpoint molecules. To explore the relationships in gene expression, the Ggcor package was employed to analyze correlations between core genes and gene families such as TNF and chemokines.

### Consensus clustering

The ConsensusClusterPlus package was utilized to do unsupervised clustering for disease subgroup mining, which is a commonly employed technique. The most suitable K-value was established by analyzing clustering heatmaps and cumulative distribution function (CDF) curves, with each sample classified according to its subgroup. The FactoMineR package and factoextra package were utilized to generate PCA diagrams, while the pheatmap tool was employed to visualize the expression heatmap of core genes in various subgroups.

### Drug screening and transcription factor analysis

Drug predictions were conducted using Enrichr’s DSigDB database (https://maayanlab.cloud/Enrichr/), and the ggplot2 software was utilized to display the top 10 drugs. Transcription factor predictions were conducted utilizing the ChEA3 website (https://maayanlab.cloud/chea3/), and the Cytoscape software was used to generate a network diagram illustrating the relationships between medicines, transcription factors, and their respective target genes.

### Culture and treatment of ATDC5 cells

ATDC5 mouse chondrocytes (SNL-178; Sunncell, Wuhan, China) were cultured in the DMEM/F12 (Gibco) with 5% FBS. The ATDC5 cell line has been validated through STR profiling and recent mycoplasma testing. All cells were maintained in a humidified incubator that contained 5% CO2 at 37°C. Interleukin-1β (IL-1β) can induce cartilage degradation by promoting the expression of matrix metalloproteinases (MMPs) in chondrocytes (29) and is widely used in the inflammatory induction model of chondrocytes in OA (30). We used IL-1β to stimulate ATDC5 cells to simulate this microenvironment of inflammation and used qRT-PCR to verify the expression level of related genes. The cells were seeded onto 96-well plates with 1,0000 cells per well and cultivated with IL-1β to simulate the cell variation observed in OA.

### RNA extraction and quantitative real-time polymerase chain reaction

The extraction of total RNA from ATDC5 cells was performed using the EZ-press RNA Purification Kit (EZBioscience). The quantification of RNA was carried out using the NanoDrop 2000 spectrophotometer (Thermo, Waltham, USA). Reverse transcription was performed using a Reverse Transcription Master Mix (EZBioscience) to produce complementary DNA. The qPCR assay was conducted using Vazyme buffers on QuantStudio 7 (Thermo). The expression levels were normalized using b actin as a reference. The primers are listed in **Table 1**.

**Table 1:** Primer sequences for qRT-PCR analysis.

Name	Origin	Forward (5’→3’)	Reverse (5’→3’)
Col2a1	Mouse	GGGAATGTCCTCTGCGATGAC	GAAGGGGATCTCGGGGTTG
Sox9	Mouse	CGGAACAGACTCACATCTCTCC	GCTTGCACGTCGGTTTTGG
Mmp3	Mouse	ACATGGAGACTTTGTCCCTTTTG	TTGGCTGAGTGGTAGAGTCCC
Mmp13	Mouse	CTTCTTCTTGTTGAGCTGGACTC	CTGTGGAGGTCACTGTAGACT
WDR74	Mouse	TCGGCAAGGGCGTCTACTGG	GGGGCTTTGAAGGGTGACACTG
TNFRSF12A	Mouse	GGGGCTTTGAAGGGTGACACTG	CGCCAAAACCAGGACCAGACTAAG
β-actin	Mouse	GGCTGTATTCCCCTCCATCG	CCAGTTGGTAACAATGCCATGT

### Immunohistochemistry assays

Tissues were fixed in 4% paraformaldehyde (PFA) and subsequently decalcified with ethylenediaminetetraacetic acid (EDTA, pH 7.4). The tissues were processed for paraffin embedding and cut into slices with a thickness of 4 µm. For immunohistochemistry (IHC) staining of human tissues, the sections were subjected to heat at 95°C for 15 minutes, followed by treatment with 3% H2O2 and 0.5% Triton X-100. The 10% bovine serum albumin was used to inhibit nonspecific binding for one hour at room temperature. The sections were subsequently subjected to an overnight incubation at 4°C with the primary antibody (WDR74, 1:200, GeneTex, GTX119013) and (TNFRSF12A, 1:100, Affinity Biosciences, DF8023). Finally, the sections were subjected to incubation with a secondary antibody, followed by counterstaining with hematoxylin and visualization with DAB solution for IHC. The level of immunoreactivity was quantified using Image J software.

### Statistical analysis

Data are presented as means with standard error (SD) to account for variability within our datasets. A Student’s t-test was used to compare the differences between the two groups. This method is appropriate for our experimental design and is a standard approach for comparing means in independent samples. For comparisons involving more than two groups, we used one-way ANOVA followed by *post-hoc* tests to adjust for multiple comparisons, ensuring that the results were robust and reliable. The Mann-Whitney U test was utilized for non-parametric data to compare differences between groups. Pearson’s correlation coefficient was calculated to assess the relationship between continuous variables. Linear regression models were used to evaluate the association between variables, adjusting for potential confounding factors. A P value of less than 0.05 was considered statistically significant for all tests, which is a conventional threshold in many scientific fields. All statistical analyses were conducted using Prism GraphPad, SPSS (version 24.0), and R program (version 3.6.2), which are widely recognized and validated tools in the field.

## Results

### Cell composition and characteristics in OA

**Figure 1:**
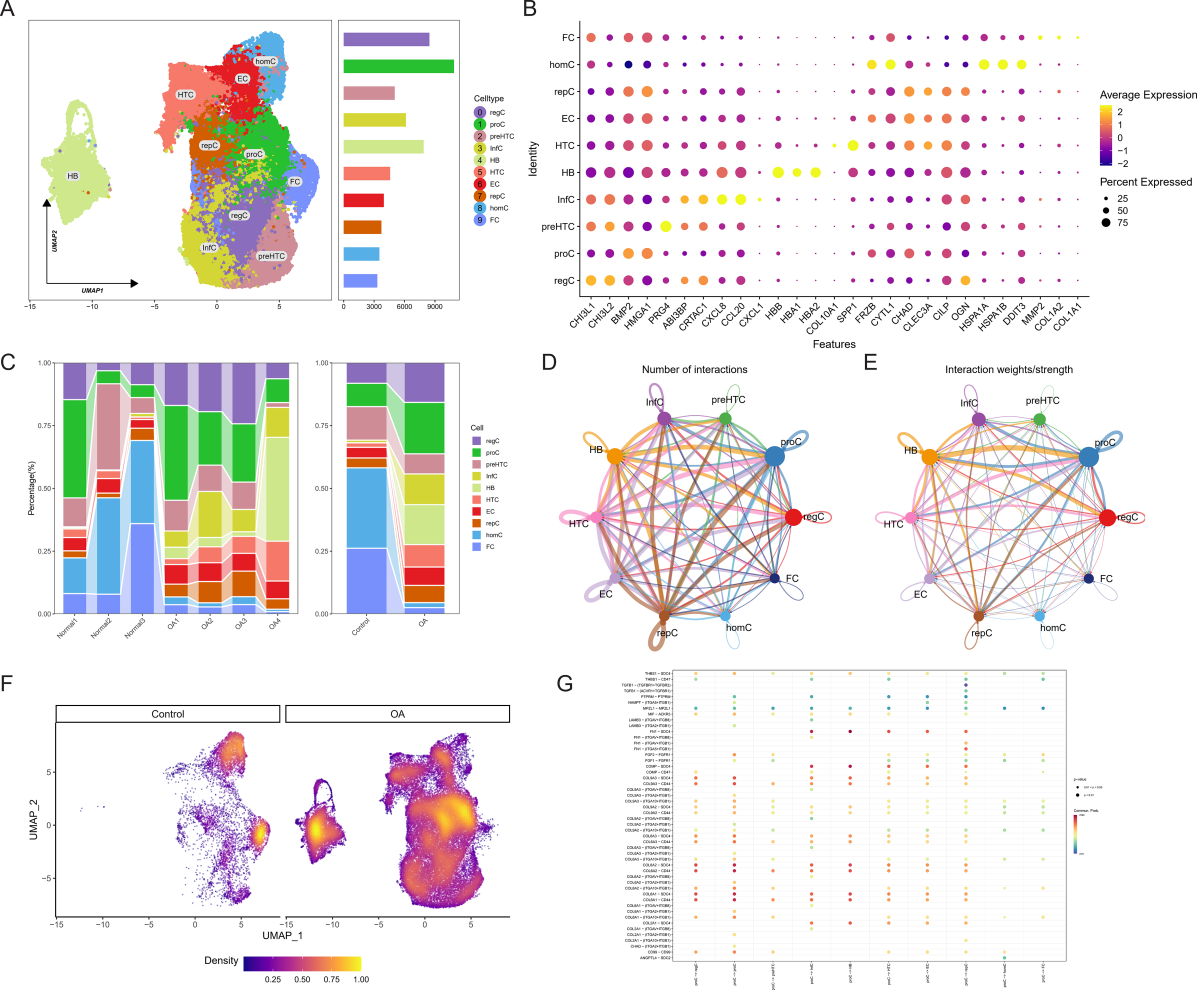
Identification of osteoarthritis (OA) cartilage cell composition and relationships through single-cell sequencing. **(A)** UMAP plot depicting the clustering of cells into 10 groups. **(B)** Bubble chart illustrating marker genes associated with each cell type. **(C)** Stacked bar graph showing the proportions of cell types. **(D, E)** CellChat communication analysis elucidating the quantity and intensity of cell-to-cell communication. **(F)** Density plot displaying the core cell type composition in control and OA groups. **(G)** Bubble chart indicating receptor-ligand pairs involved in cell communication interactions.

Following the integration and quality verification of the single-cell data, a total of 7924 cells from the normal group and 4955 cells from the OA group were selected for further study. According to the ElbowPlot analysis, the top 15 primary components were chosen to reduce the number of dimensions and group the data. This led to the formation of 15 clusters, numbered from 0 to 14. The clusters were categorized into 10 cell types based on SingleR analysis and information from existing literature (**Figure 1A**). The marker genes associated with each cell type are as follows: homeostatic chondrocytes (HomC) (HSPA1A, HSPA1B, HSPA6), regulatory chondrocytes (RegC) (CHI3L1, CHI3L2), proliferating chondrocytes (ProC) (BMP2, HMGA1), prefibrocartilage chondrocytes (preHTC) (PRG4, ABI3BP, CRTAC1), inflammatory chondrocytes (InfC) (CXCL8, CCL20, CXCL1), red blood cells (HBB, HBA1, HBA2), hypertrophic chondrocytes (HTC) (COL10A1, SPP1), effector chondrocytes (EC) (FRZB, CYTL1, CHAD, CLEC3A), repair chondrocytes (RepC) (CILP, OGN), and fibrocartilage chondrocytes (FC) (MMP2, COL1A2, COL1A1) (**Figure 1B**). The stacked bar and density plots of cell proportions illustrate a significant increase in the proportions of RegC, ProC, InfC, and HTC in the OA group, along with a substantial decrease in HomC and FC (**Figures 1C, D**). The analysis of cell communication reveals that ProC demonstrates a significant number of output signals, suggesting its potential involvement in the advancement of OA (**Figures 1D, E**). **Figure 1F** presents a bubble chart that shows the receptor-ligand pairings involved in ProC communication. This chart also demonstrates the activation of several pathways associated with collagen. Plasma cells showed the highest ubiquitination score, as determined by the AUCell algorithm (**Figure 1G**).

### Ubiquitination activity and core gene identification in OA

**Figure 2:**
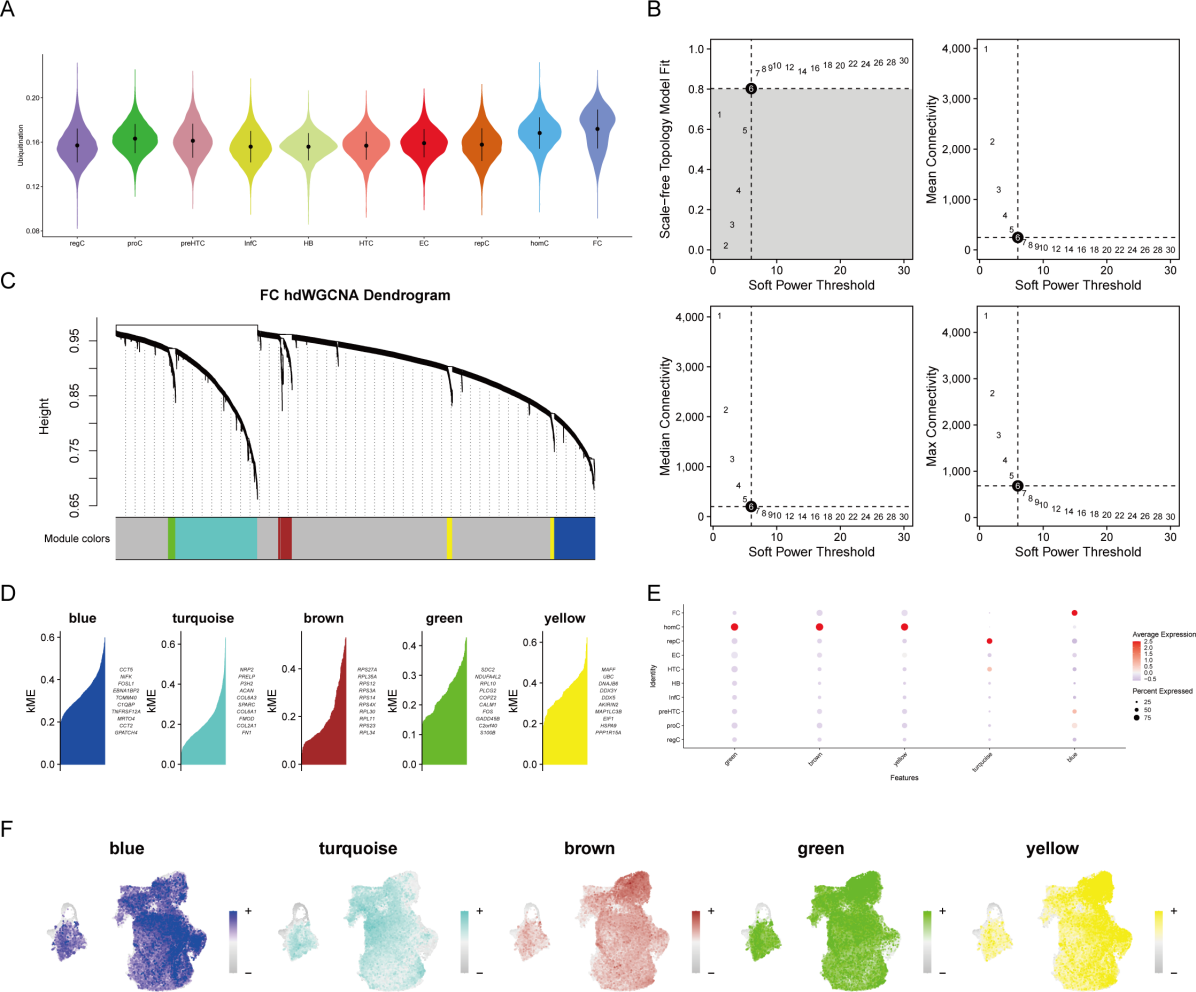
High-dimensional weighted gene co-expression network analysis (hdWGCNA) of single-cell data. **(A)** Ubiquitination scoring to identify ubiquitination-associated cell types. **(B)** Soft threshold selection plot to determine the optimal threshold. **(C)** Gene module clustering diagram. **(D–F)** Identification of specific gene modules and their correlation analysis with cell types, highlighting gene clusters most relevant to cell types.

The study examined which cell type exhibited an upregulation of ubiquitination activity following a preliminary analysis of the cellular composition and characteristics in OA. The AUCell algorithm was employed to calculate the ubiquitination scores of each cell type, which were composed of the ubiquitination-related genes. FC cells, as evidenced by their highest score, were involved in the most ubiquitination processes (**Figure 2A**). The hdWGCNA algorithm was employed to identify core genes associated with ubiquitination. A flexible threshold of 6 was selected to divide the genes into five functional modules: blue, turquoise, brown, green, and yellow, as determined by the FC’s characteristics (**Figures 2B–D**). FC has the highest correlation with genes in the blue module, as evidenced by a correlation bubble chart (**Figure 2E**). This suggests that these genes may be associated with the ubiquitination process. The feature plot also suggests that the aggregate scores of genes in the blue module are concentrated in FC (**Figure 2F**).

**Figure 3:**
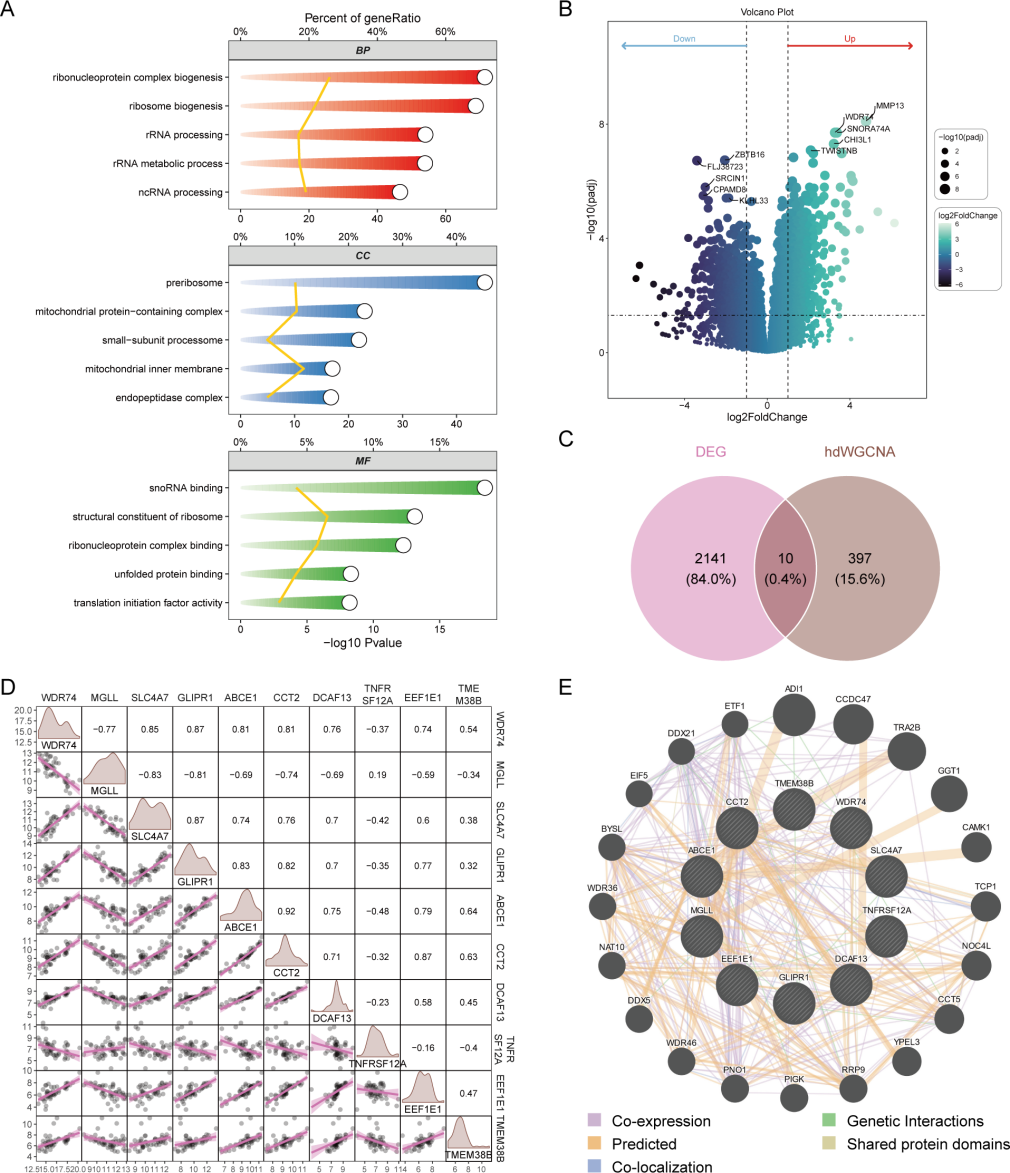
Screening of differentially expressed ubiquitination-related genes. **(A)** GO enrichment analysis of genes in the blue module. **(B)** Volcano plot from differential expression analysis of OA dataset, showcasing differentially expressed genes. **(C)** Venn diagram identifying OA ubiquitination-related genes from bulk sequencing data. **(D)** Correlation analysis of differentially expressed ubiquitination-related genes. **(E)** GENEMANIA analysis of protein-protein interaction networks of differentially expressed ubiquitination-related genes.

The blue module contained 407 genes with a correlation greater than 0.3. Enrichment analysis revealed that these genes are primarily associated with the synthesis of ribosomal RNA and non-coding RNA (**Figure 3A**). The bulk sequencing data of OA was also screened for corresponding OA-related genes, resulting in 2151 differentially expressed genes (**Figure 3B**). A Venn diagram of the core genes in the blue module and the differential genes from bulk sequencing identified 10 OA ubiquitination-related genes (**Figure 3C**). Expression correlation analysis demonstrated that the majority of genes are positively correlated, with the exception of MGLL, which is negatively correlated with other genes (**Figure 3D**). GENEMANIA analysis indicates that OA ubiquitination-related genes primarily have predicted functions (**Figure 3E**).

### Diagnostic performance of core OA-related ubiquitination-related genes

**Figure 4:**
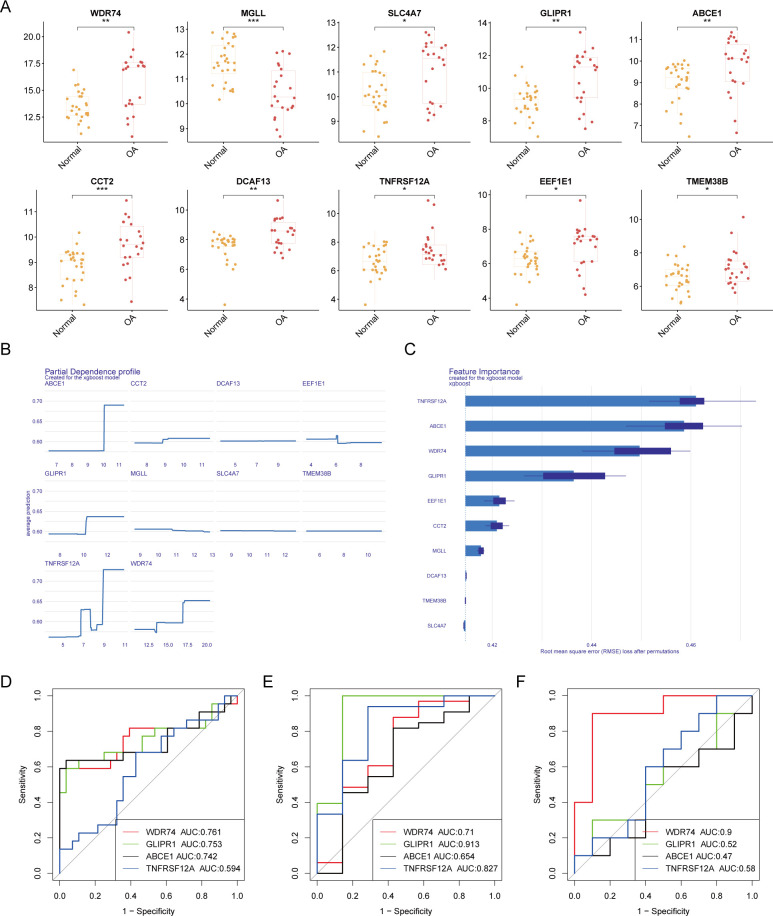
Machine learning-based selection of core ubiquitination-related genes. **(A)** Box plots of differential expression for ubiquitination-related genes. **(B, C)** XGBoost machine learning algorithm for the selection of core ubiquitination-related genes. **(D–F)** ROC curves of individual core ubiquitination-related genes across three validation cohorts.

**Figure 5:**
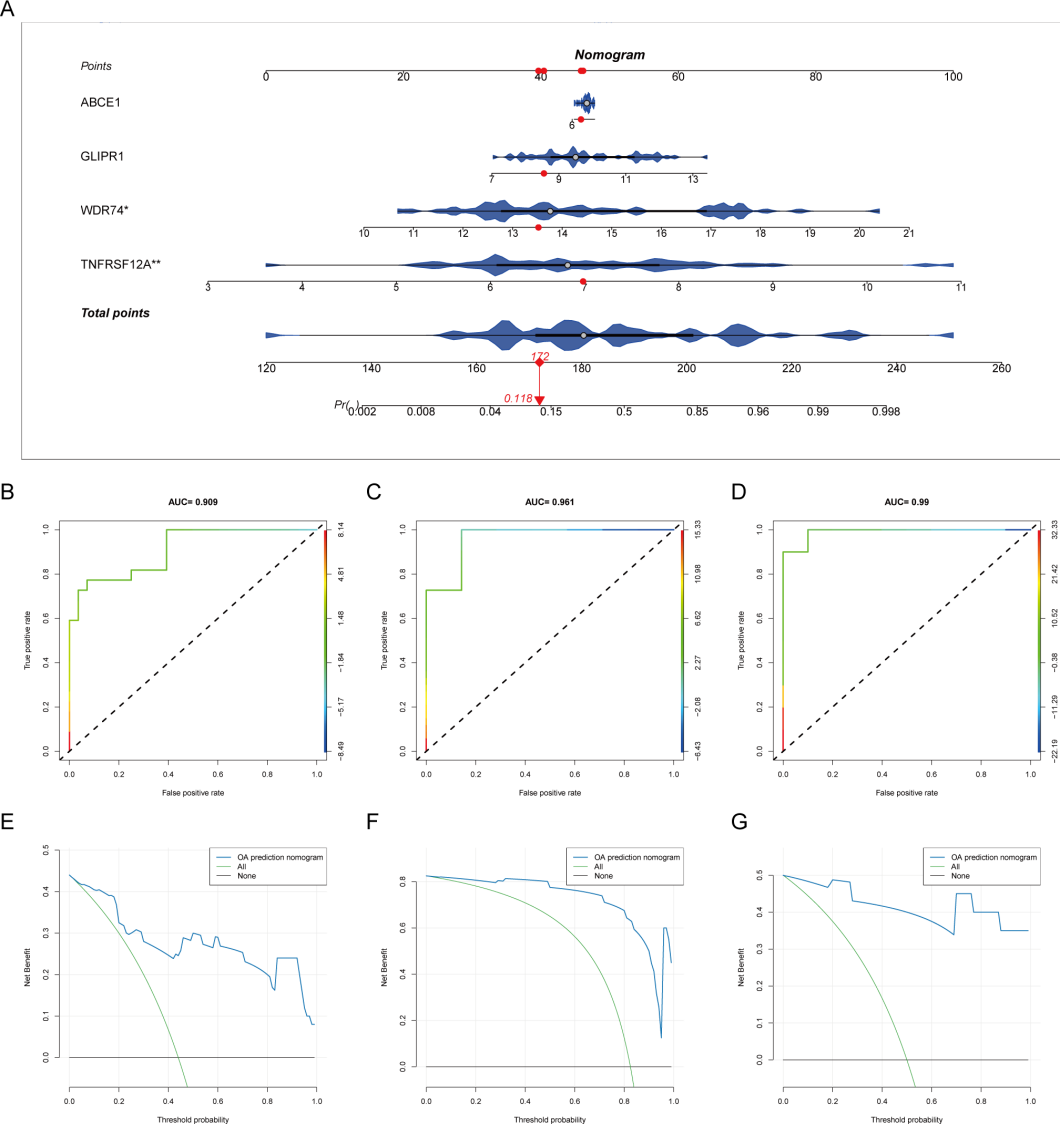
Diagnostic model of core ubiquitination-related genes. **(A)** Nomogram of the diagnostic model for core ubiquitination-related genes. **(B–D)** ROC curves of the diagnostic model in different OA datasets. **(E–G)** Decision curve analysis (DCA) of the diagnostic model across various OA datasets.

Box plots of differential expression show that, except for MGLL, other OA ubiquitination-related genes are highly expressed in the OA group (**Figure 4A**). The XGBoost algorithm calculated the partial dependence and feature importance of the 10 OA ubiquitination-related genes, and based on the decrease in feature importance, TNFRSF12A, ABCE1, WDR74, and GLIPR1 were identified as core OA ubiquitination-related genes (**Figures 4B, C**). The diagnostic efficacy of individual core OA ubiquitination-related genes was unstable, with AUC values fluctuating between 0.5 and 0.9 in the training and two validation sets (**Figures 4D–F**). To enhance the diagnostic capability of the core OA ubiquitination-related genes, a diagnostic model was constructed, with WDR74 and TNFRSF12A emerging as independent diagnostic predictors (**Figure 5A**). Both the training set and the two validation sets demonstrated excellent diagnostic ability, with AUC values greater than 0.9 (**Figures 5B–D**). Decision curves also indicated that the diagnostic model aids in clinical decision-making (**Figures 5E–G**).

### Immunological relevance and subgroup stratification

**Figure 6:**
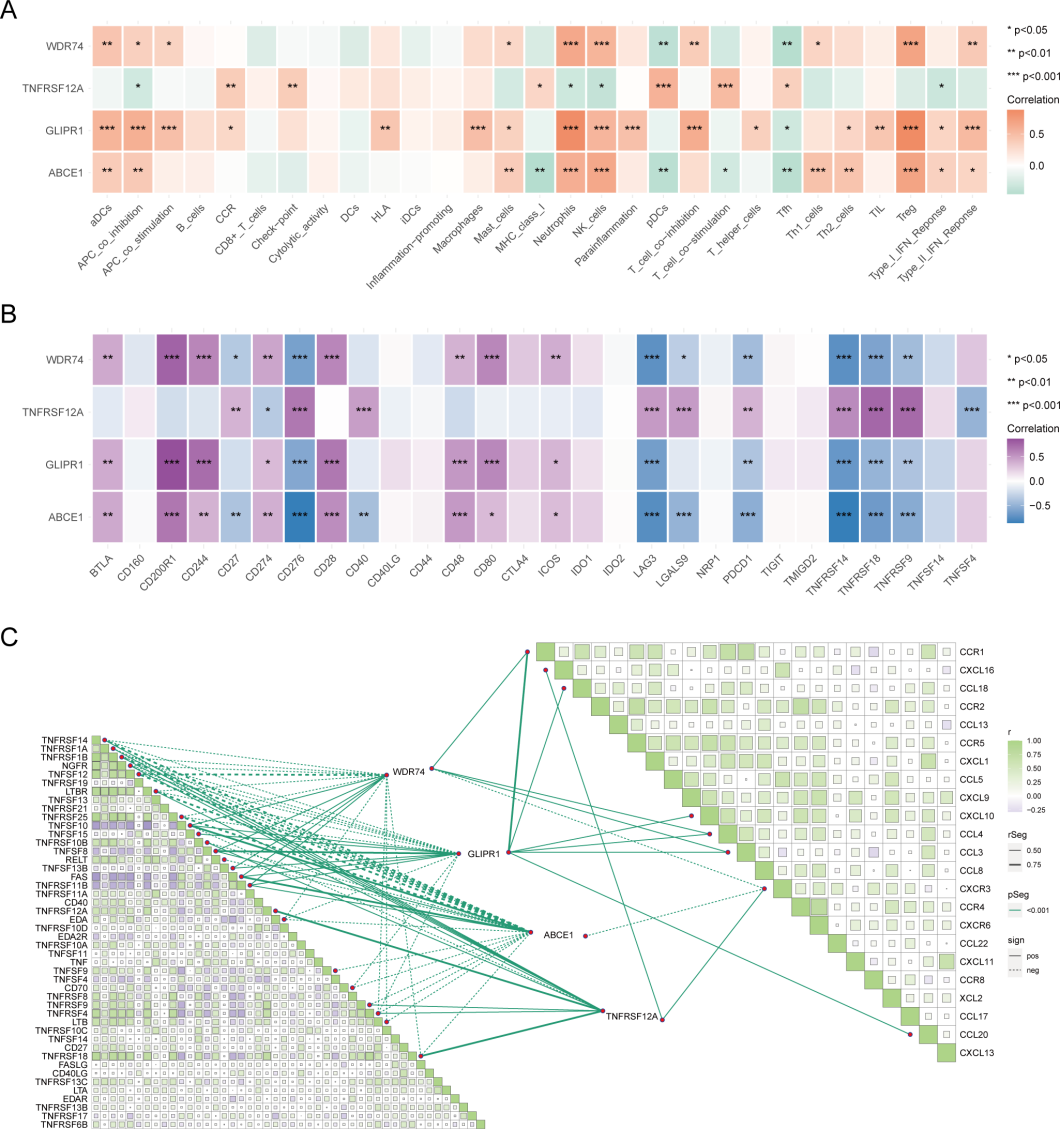
Immunological relevance of core ubiquitination-related genes. **(A)** Heatmap of the correlation between core ubiquitination-related genes and immune cells and processes. **(B)** Heatmap illustrating the correlation between core ubiquitination-related genes and immune checkpoint molecules. **(C)** Correlation matrix showing the association between core ubiquitination-related genes and chemokine and TNF families.

**Figure 7:**
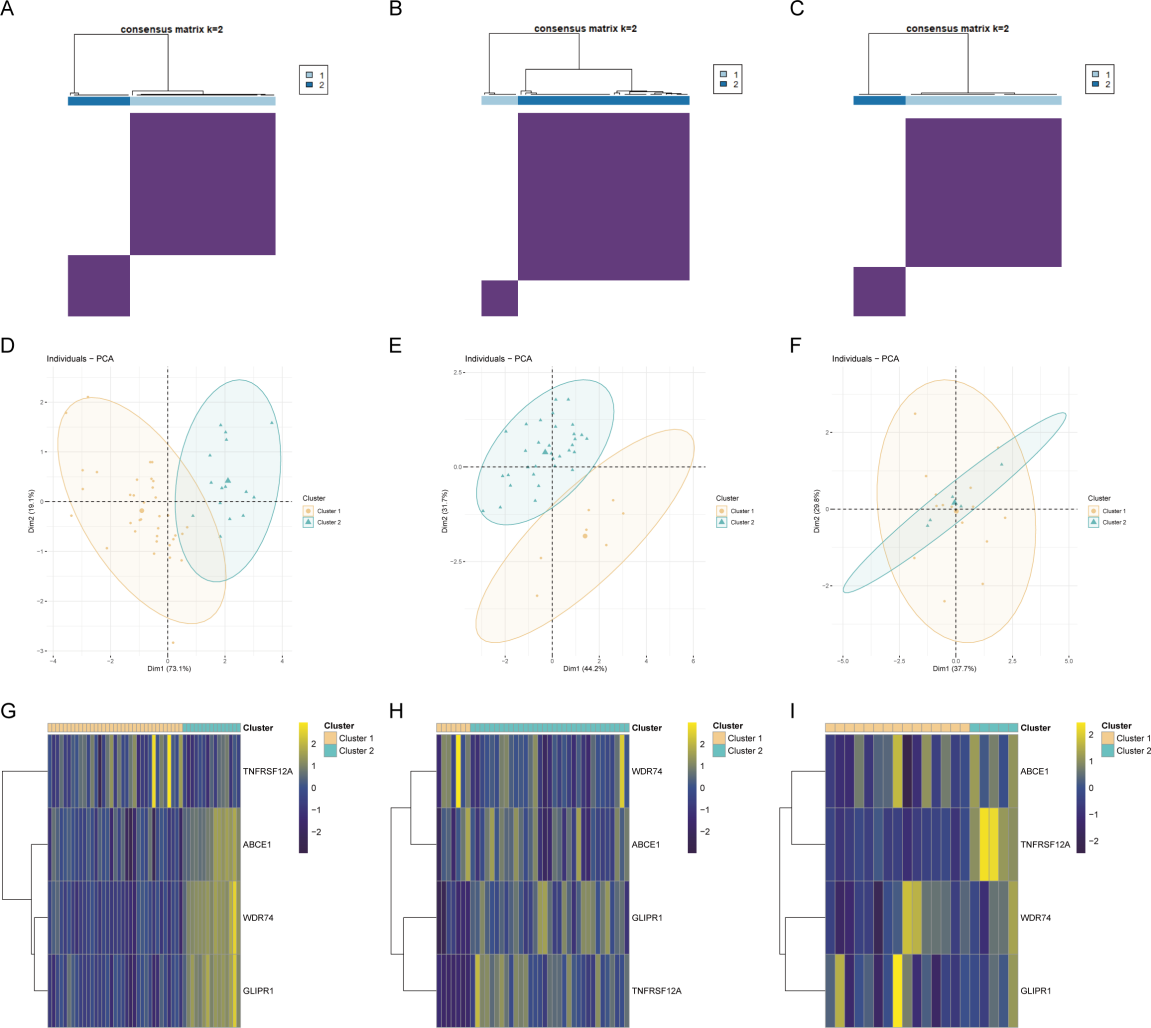
Construction of ubiquitination-related molecular subtypes. **(A–C)** Consensus clustering analysis to identify molecular subtypes in different OA datasets. **(D–F)** PCA analysis based on molecular subtypes. **(G–I)** Expression heatmaps of core ubiquitination-related genes across identified molecular subtypes.

Genes related to core OA ubiquitination are generally positively correlated with immune cells and immune processes, and they also exhibit an extensive positive correlation with the expression of immune checkpoints (**Figures 6A, B**). This suggests that these genes are associated with immune processes and may be potential targets for immunotherapy. The correlation matrix indicates that the expression of core OA ubiquitination-related genes is significantly correlated with the TNF and chemokine families. This suggests that the pathogenesis of OA may be associated with inflammatory immune processes, with ubiquitination potentially being implicated (**Figure 6C**). Furthermore, genes associated with the ubiquitination of core OA also hold clinical subgroup guiding importance. Consensus clustering analysis reveals that both the training and validation sets of patients can be classified into two distinct subgroups based on the expression patterns of core OA ubiquitination-related genes (**Figures 7A–C**). Furthermore, the principal components on the principal component analysis (PCA) plot are significantly separated for these subgroups (**Figures 7D–F**). In the same way, the two subgroups also show variation in the expression of key genes linked to OA ubiquitination. Specifically, ABCE1, WDR74, and GLIPR1 tend to exhibit comparable expression patterns (**Figures 7G–I**).

### Drug target prediction and transcription factor analysis

**Figure 8:**
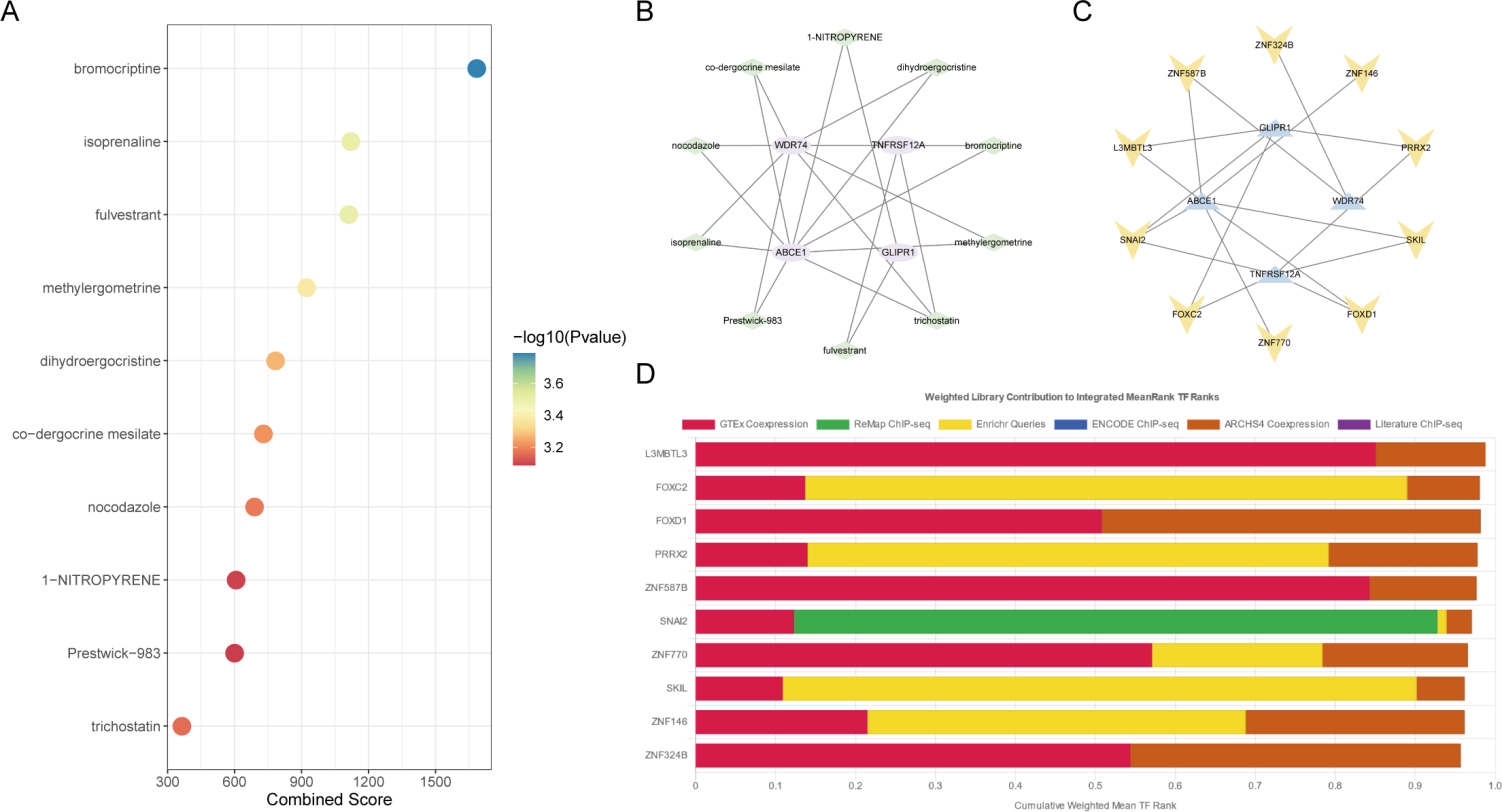
Drug and transcription factor analysis of core ubiquitination-related genes. **(A, B)** Drug screening based on the DSigDB database. **(C, D)** Prediction of transcription factors regulating core ubiquitination-related genes using the ChEA3 database.

The target drugs for core OA ubiquitination-related genes were predicted, showing the top ten drugs and their regulatory gene networks (**Figures 8A, B**). In addition, the regulatory transcription factors for core OA ubiquitination-related genes, including FOXD1, were also predicted (**Figures 8C, D**).

### The expression level of WDR74 and TNFRSF12A

The diagnostic model demonstrated that WDR74 and TNFRSF12A were independent diagnostic predictors, and both the training set and the two validation sets exhibited exceptional diagnostic ability. Consequently, the study selected WDR74 and TNFRSF12A for external validation in order to investigate their relationship with OA.

**Figure 9:**
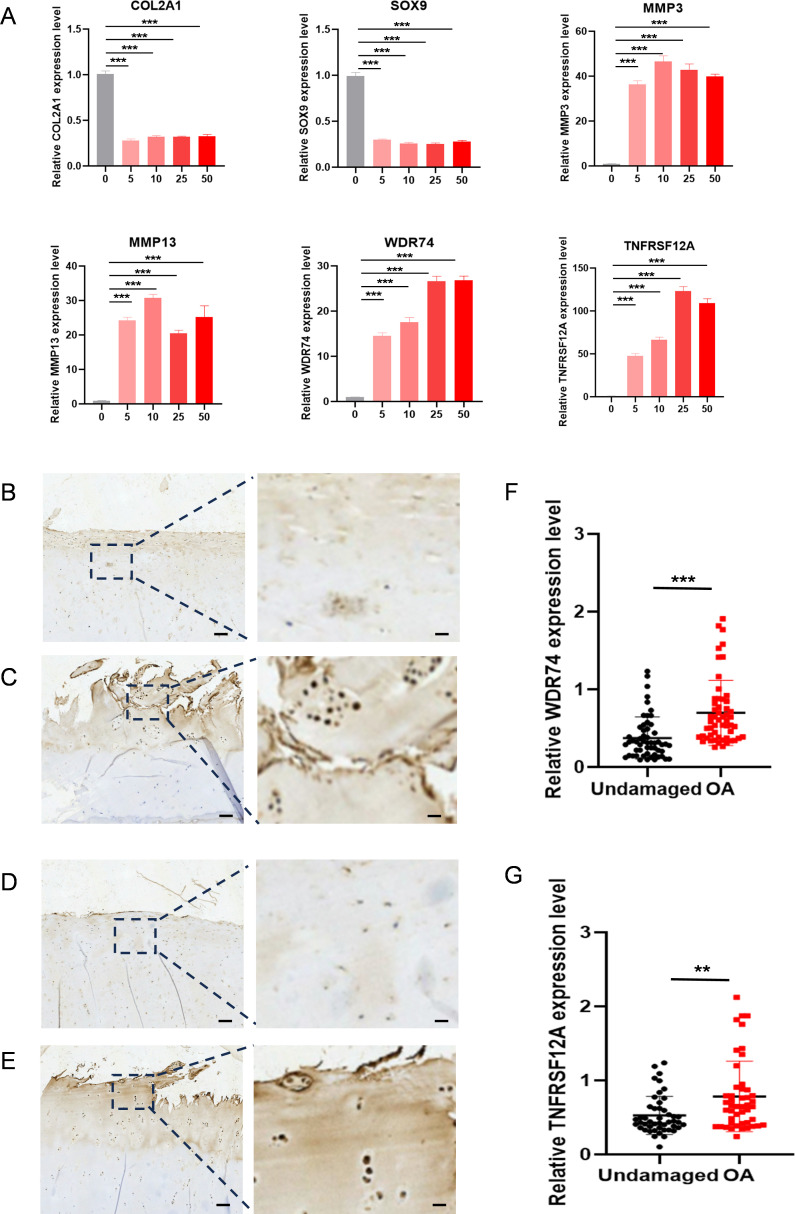
The expression level WDR74 and TNFRSF12A in ATDC5 cells with different concentrated IL-1β and human OA cartilage. **(A)** qRT-PCR measurement of Col2a1, Sox9, Mmp3, Mmp13, WDR74 and TNFRSF12A in ATDC5 cells treated with different concentrated IL-1β. ** p<0.01; *** p<0.001. **(B, C)** Immunohistochemistry assay with anti-WDR74 in undamaged cartilage tissues and OA cartilage tissues. **(D, E)** Immunohistochemistry assay with anti-TNFRSF12A in undamaged cartilage tissues and OA cartilage tissues. Scale bar, Left, 100μm; Right, 20μm. **(F, G)** Relative expression level in OA (n=55) and corresponding undamaged (n=55) cartilage tissues based on an immunohistochemistry assay and significance was evaluated by paired Student’s t test.

IL-1β decreased the expression of Col2a1 and SOX9 in ATDC5 cells and increased the expression of Mmp3 and Mmp13, indicating that induction methods were consistent with the molecular characteristics of OA. At the same time, IL-1β can also upregulate the expression of WDR74 and TNFRSF12A (**Figure 9A**). Our results showed that WDR74 and TNFRSF12A genes were significantly upregulated in the OA group, which was confirmed by external human samples (**Figures 9B–E**). Immunohistochemical analysis showed that WDR74 and TNFRSF12A were significantly upregulated in OA, suggesting a correlation between these genes and OA (**Figures 9F, G**).

## Discussion

OA is a multifactorial degenerative joint disease characterized by the progressive deterioration of articular cartilage, subchondral bone remodeling, and synovial inflammation (31, 32). So far, no singular definitive cause of OA has been established. The primary risk factors for OA are aging and joint injury, both of which can result in cartilage deterioration (33). This deterioration is partially attributable to the formation of senescent chondrocytes in joint tissues, which frequently display distinct cellular dysfunctions, such as autophagy downregulation, mitochondrial impairment, and inflammasome overactivation (14, 34). Nonetheless, the precise mechanisms by which these risk factors induce the start of OA are not fully comprehended, and the molecular processes involved remain ambiguous. To enhance comprehension of the pathogenic mechanisms of OA, novel diagnostic tools, strategies, and technologies must be utilized to investigate its pathogenesis.

Recent studies have emphasized the pivotal function of ubiquitination in modulating diverse cellular processes related to OA (35). Ubiquitination is a crucial post-translational modification that plays a role in protein degradation, cell cycle regulation, and apoptosis, all of which are vital for preserving the homeostasis of bone joint tissues. The dysregulation of ubiquitination pathways is strongly associated with the pathophysiology of OA, as changes in protein turnover can result in the accumulation of misfolded proteins and the disruption of normal cellular activities (36). In OA, articular chondrocytes inevitably experience metabolic imbalance, leading to the degradation of the cartilage matrix. Ubiquitination regulates this process by mediating the degradation of specific proteins. For instance, the ubiquitination of certain cytokines and enzymes may enhance or inhibit chondrocyte apoptosis, thereby affecting cartilage health (37). Additionally, ubiquitination plays a crucial role in the chronic inflammatory process of OA, as it impacts inflammation-related signaling pathways (such as the NF-κB pathway) and subsequently regulates the expression of pro-inflammatory cytokines. The excessive expression of these cytokines can result in damage and pathological changes to joint tissues (35). Moreover, ubiquitination is involved in regulating the life and death decisions of chondrocytes. By modulating the ubiquitination of apoptosis-related proteins (such as p53 and Bcl-2), the balance between cell survival and death is disrupted, further promoting cartilage degradation (38). Furthermore, ubiquitination can influence the activity of transcription factors, thereby regulating the expression of OA-related genes (39). For example, the ubiquitination of certain transcription factors may lead to their degradation in the nucleus, affecting the expression of target genes (such as matrix metalloproteinases, MMPs), which play a critical role in the degradation of the cartilage matrix (40). Therefore, ubiquitination exerts multifaceted effects on the occurrence and progression of OA, impacting chondrocyte function, the inflammatory state of the joints, and the survival and apoptosis of cells.

Comprehending the interaction between ubiquitination-related genes and OA yields significant insights into the molecular mechanisms underlying disease progression and presents new opportunities for prospective treatment approaches. In the study, we revealed a diagnostic model for OA by integrating URG expression with ML algorithms, followed by qRT-PCR and IHC validation in gene levels and on human specimens, respectively. The identification of WDR74 and TNFRSF12A as core biomarkers underscored their potential as therapeutic targets and highlighted the value of precision medicine in OA management. Our approach diverged from traditional diagnostic methods by harnessing the power of ML to analyze the complex data generated from scRNA-seq. The use of ML algorithms has allowed for the identification of subtle patterns in URG expression that are indicative of OA, surpassing the limitations of conventional statistical methods (41).

In this combined investigation, of ML and bioinformatics, we explored cell-to-cell communication within the joint microenvironment using the CellChat program. This research revealed complex networks of cellular interactions that may be potentially pivotal in the development of OA. Notably, proliferating chondrocytes (ProC), identified as significant communicative signal producers, may play a critical role in driving disease progression, warranting further investigation. Utilization of the AUCell program facilitated the calculation of ubiquitination scores across diverse cell subpopulations, crucial for identifying URGs with prominent ubiquitination activity. Employing hdWGCNA, we identified specific cell types and gene modules closely associated with OA. The blue module, notably linked with FC cells, is particularly notable for its potential involvement in ubiquitination processes. Differential expression analysis using Limma, complemented by enrichment analysis via clusterProfiler, highlighted a subset of URGs showing differential expression in OA. These genes, implicated in roles such as ribosomal RNA and non-coding RNA synthesis, likely contribute to OA’s molecular pathogenesis (42, 43). Integration with bulk RNA sequencing data facilitated the identification of a core set of OA-related URGs, subsequently subjected to additional ML-based analysis to refine their significance in disease mechanisms.

Notably, our research identified core URGs linked to OA, especially WDR74, and TNFRSF12A, laying a solid foundation for the development of important biomarkers for early OA detection. Our findings demonstrated that these two genes are independent predictors of OA diagnosis, exhibiting excellent diagnostic efficacy in the training cohort. Their predictive value has been validated in two independent cohorts and further confirmed through histological analysis.

The involvement of WDR74 in OA is intricate and requires a comprehensive analysis within the context of molecular biology, genetics, and clinical significance. The WDR74 gene encodes the WD repeat domain 74 protein and is implicated in different functions of cartilage cells, especially in relation to OA, a degenerative joint disorder marked by the progressive degradation of cartilage (44). WDR74 is a key component of the MTR4-EXO1 complex, which is involved in the degradation of pre-ribosomal RNA in the nucleolus (45). This process is essential for ribosome biogenesis and protein synthesis, both of which are crucial for maintaining cartilage integrity in chondrocytes (46). Dysregulation of WDR74 could disrupt this process, potentially leading to the accumulation of misfolded proteins and the disruption of normal cellular activities, both of which are associated with OA pathogenesis (36). Moreover, WDR74 has been shown in the regulation of the Wnt/β-catenin signaling pathway, which plays a vital role in chondrocyte proliferation and differentiation (47). Dysregulation of this pathway can lead to the degeneration of articular cartilage, a hallmark of OA (48). Furthermore, WDR74’s role in ribosomal activity could indirectly impact cartilage health, as chondrocytes in the joint are metabolically active and rely on efficient protein synthesis to maintain cartilage integrity (49).

TNFRSF12A, encoding the TNF-like weak inducer of apoptosis (TWEAK) receptor r, is part of the TNF receptor superfamily and regulates cell survival, proliferation, and apoptosis (50). Studies suggest that during OA progression, dysregulation of mechanotransduction and extracellular matrix pathways may be influenced by TNFRSF12A (51). Additionally, TNFRSF12A has been identified as a potential candidate gene driving pathological calcification in OA cartilage, contributing to disease progression and irreversibility (52). The TNFRSF12A-TWEAK pathway serves as a central mediator of inflammatory responses and regulates the expression of catabolic factors like matrix metalloproteinases (MMPs) and ADAMTS enzymes, which are important in breaking down the cartilage matrix (53). The activation of this pathway can lead to increased joint inflammation and the deterioration of cartilage in OA (54). Furthermore, the TWEAK-Fn14 axis could potentially play a role in the development of OA by influencing the survival and function of chondrocytes, with TWEAK promoting the production of pro-inflammatory cytokines such as IL-6 and TNF- α by different cell types present in the joint (55).

Long-term low-level chronic inflammation and innate and adaptive immune system activation play critical roles in all aspects of OA pathogenesis (56, 57). The results of our study indicate a strong connection between genes related to ubiquitination in OA and immune cells, indicating a complex interplay between the immune system and OA pathogenesis. This paves the way for potential immunotherapeutic approaches that could utilize immune response modulation to treat or slow the progression of OA. Additionally, the correlation between these genes and the TNF and chemokine families suggests the involvement of inflammation in OA, where ubiquitination processes may play a role. The significance of our findings lies in the ability to categorize patients into specific subgroups based on the expression of key OA ubiquitination-related genes. The categorization has the potential to provide valuable insights for tailoring treatment tactics to individual patients and enhancing patient outcomes. Moreover, the identification of therapeutic targets and regulatory transcription factors for these essential genes provides opportunities for the advancement of precision medicines.

Although the precise roles of WDR74 and TNFRSF12A in OA are still being explored, their participation in critical cellular processes that influence cartilage integrity positions them as promising candidates for further research and therapeutic development. Comprehending the mechanisms by which WDR74 and TNFRSF12A contribute to OA pathogenesis is essential for evaluating their potential as therapeutic targets. Such insights could facilitate the development of innovative treatment strategies for individuals afflicted by this severe ailment.

There are some limitations in our study. Firstly, the generalizability of our diagnostic model needs validation in larger cohorts with more diverse demographic characteristics. This will help to ensure that our findings are applicable across different populations. Secondly, while we have identified WDR74 and TNFRSF12A as potential biomarkers for early OA, the functional significance of these genes in the context of OA pathogenesis requires further experimental validation. Additional *in vitro* and *in vivo* studies are necessary to confirm their roles in disease development and progression. Lastly, potential biases in our study may arise from the reliance on existing datasets, which may not fully capture the heterogeneity of OA across different patient populations. To address this, we have implemented rigorous quality control measures and employed advanced bioinformatics techniques to ensure the robustness of our findings. Despite these limitations, our study provides valuable insights into the role of ubiquitination-related genes in OA and lays the groundwork for future research in this area.

## Conclusion

In conclusion, our study has identified WDR74 and TNFRSF12A as potential biomarkers for early OA using machine learning and immunohistochemistry. These findings provide valuable insights into the molecular mechanisms underlying OA and may pave the way for the development of novel diagnostic and therapeutic strategies. The high diagnostic accuracy of our model, along with the validation of WDR74 and TNFRSF12A in human samples, underscores the potential clinical utility of these biomarkers. Further research is needed to fully elucidate their roles in OA pathogenesis and to translate these findings into clinical practice.

## Data Availability

The datasets generated and/or analyzed during the current study are available from the corresponding author upon reasonable request.
